# Determinants of dentists’ readiness to assess HPV risk and recommend immunization: A transtheoretical model of change-based cross-sectional study of Ontario dentists

**DOI:** 10.1371/journal.pone.0247043

**Published:** 2021-02-17

**Authors:** Musfer Aldossri, Chimere Okoronkwo, Virginia Dodd, Heather Manson, Sonica Singhal

**Affiliations:** 1 Faculty of Dentistry, Discipline of Dental Public Health, University of Toronto, Ontario, Canada; 2 Department of Periodontics and Community Dentistry, College of Dentistry, King Saud University, Riyadh, Saudi Arabia; 3 Health and Social Services Division, Haldimand and Norfolk Counties, Simcoe, Ontario, Canada; 4 Northern Ontario School of Medicine, Lakehead University, Thunder Bay, Ontario, Canada; 5 Department of Community Dentistry and Behavioral Science, University of Florida, Gainesville, Florida, United States of America; 6 Health Promotion, Chronic Disease and Injury Prevention department, Public Health Ontario, Toronto, Canada; 7 Dalla Lana School of Public Health, University of Toronto, Ontario, Canada; Federal University of Sergipe, BRAZIL

## Abstract

**Objectives:**

To evaluate dentists’ readiness to assess the history of human papilloma virus (HPV) infections and recommend immunization among their patients.

**Materials and methods:**

A link to a self-administered questionnaire was emailed to Ontario dentists. Dentists’ readiness and its determinants were assessed based on Transtheoretical Model’s ‘stages’ and ‘processes’ of change, respectively. Based on their current practices, dentists were either assigned to ‘pre-action’ or ‘action+’ stages.

**Results:**

Of the 9,975 dentists contacted, 932 completed the survey; 51.9% participants were in action stage to assess the history of HPV infections and 20.5% to recommend immunization. Internationally-trained and those whose office’s physical layout was not a concern to discuss patients’ sexual history were more likely to assess the history. Dentists with higher knowledge about HPV vaccines, not concerned about the HPV vaccine safety, comfortable discussing sex-related topics with patients, or willing to exceed their scope of practice were more ready to recommend HPV immunization to their patients.

**Conclusion:**

Improving Ontario dentists’ knowledge and communication skills and changing their self-perceived role regarding HPV infections and vaccination can increase their capacity to minimize the burden of HPV infections.

## 1. Introduction

The incidence of oral cancers in high-income countries is shifting from oral cavity cancers generally attributable to tobacco consumption to oropharyngeal cancers (OPC) linked to human papillomavirus (HPV) oral infections [1]. In Canada, for example, the 2016 Canadian Cancer Statistics Report estimated that in 2012 OPC represented 39.8% and 20.6% of new oral cancer cases diagnosed among men and women, respectively [2]. The report indicated that, in 2012, of the 3,760 newly diagnosed Canadian cancer cases, 35% were HPV-related and of those, 80% were diagnosed in men. If this trend continues, it is predicted that the incidence rates of OPC among males will surpass cervical cancer rates among females [2,3].

HPV infection is one of the most common sexually-transmitted infections, and it has been implicated as a cause of cancer for various anatomic sites including the cervix, vagina, vulva, penis, anus and oropharynx [4,5]. Vertical transmission of HPV from mother to child has also been suggested as a route of transmission, with children of HPV-positive mothers being 33.0% more likely to be HPV-positive than those of HPV-negative mothers [6]. Among HPV infections, the prevalence of HPV oral infections has been estimated as 7.7%, with incidence rate being 4.38 cases per 1000 person-months [7]. HPV infections may remain asymptomatic or may be detected as wart-like oral lesions, with most HPV oral infections tend to clear within 18 months [8]. However, a small percentage of HPV oral infections may persist for a longer period of time and transform into malignant lesion, particularly the high risk of HPV oral infections that are linked to HPV types 16 and 18 [9].

In recent decades, vaccines have been developed to prevent HPV infections and subsequent development of HPV-related cancers. Development of HPV-related OPC is associated with HPV types 16 and 18. While it is too early to identify the vaccine’s ability to lower the number of HPV-related OPC, randomized clinical trials have demonstrated the efficacy and safety of HPV vaccines [10,11]. In general, HPV vaccines are recommended for males and females ages 9–26 years [12]. In Canada, HPV-vaccine recommendations have been extended to include females ages 27–45 years [13]. While HPV vaccines are available across the globe in public or private setups, vaccine access is not consistent and the uptake and completion rates for the vaccine through school-based programs vary significantly across jurisdictions [14–18]. Therefore, attaining higher uptake of the vaccine and increased completion rates will require additional public health efforts.

Dentists are poised to contribute significantly to both the uptake of the vaccine and completion rates [19]. To improve patients’ HPV-related knowledge, some professional dental organizations encourage their members to routinely discuss the connection between HPV infection and OPC [20–22]. Dentists are also encouraged to directly recommend the vaccine to their patients [20,21]. Despite these recommendations, current literature suggests that dentists are not engaging their patients enough in discussions regarding HPV infection, available vaccines, or the need for completing the vaccine series. In Ontario, research findings indicate that only 50.0% of dentists report currently discussing the connection between HPV and OPC with all or some of their patients [23]. In U.S., among Florida dentists, survey findings indicate 9.0% of dentists report discussing the HPV immunization with some female patients [24]. These findings indicate the need for dentists to play a more active role in the prevention of HPV infections.

Understanding the determinants of dentists’ readiness to assess HPV risk and recommend the HPV immunization is crucial for increasing the presence of dental professionals in HPV prevention. Daley and colleagues identified dentists’ knowledge of HPV and the HPV vaccine, their attitudes towards discussing HPV and its relation to sex, and the barriers dentists identified are keeping them from addressing HPV infections with their patients [24,25]. Our previous research demonstrated that dentists’ readiness to assess the history of patients’ HPV infections is an important predictor for their readiness to discuss the connection between HPV and OPC [23]. However, the determinants of dentists’ readiness to assess a patient’s history of HPV infection remains unknown. Furthermore, factors influencing dentists to recommend HPV vaccine to their patients are not well understood. Despite a theoretical and behavioral basis for some determinants, larger quantitative studies are needed to confirm their role in dentists’ readiness to discuss HPV infection with patients. As such, we have no understanding in this regard about Canadian dentists.

Accordingly, the objectives of this study were to determine Canadian dentists’ readiness to: assess patient’s HPV infection history; recommend HPV immunization; and explore associated determinants.

## 2. Materials and methods

### 2.1 Ethics approval

Ethics approval was obtained for conducting this survey from the Research Ethics Board of Public Health Ontario (File number 2017–040.01). A statement was included in the survey questionnaire to record the active consent of participants. To further protect respondents’ privacy, sociodemographic groups with five or less respondents that could not be combined with another group, were dropped from the final analyses.

### 2.2 Survey instrument

The survey instrument was developed following a robust review of the literature. Based on relevancy to the study’s purpose, survey questions were framed using the Transtheoretical Model (TTM; stages of change (SOC) and processes of change (POC)) [26]. The survey was reviewed by public health experts and dental professionals including general dentists, oral pathologists and dental public health specialists to ensure its face validity; the survey was pilot-tested by ten Ontario dentists. A link to the final 25-item self-report online survey (S1 File) was emailed to the members of the Royal College of Dental Surgeons of Ontario (RCDSO, the regulatory body for Ontario dentists). The survey was administered between August 2, 2017 and August 30, 2017, with two reminders after two and three weeks. The detailed description of survey development has been previously published [23].

Dentists’ readiness to assess HPV infection history/status and recommend HPV immunization and the associated determinats were assessed based on the TTM [26]. Theoretically, readiness to act (assess HPV infection history/status and recommend HPV immunization) can be viewed as the gap between a current behavioral practice and the target behavior. The TTM assesses this gap by assigning individuals, based on their current behavior, to one of the six SOC, namely precontemplation, contemplation, preparation, action, maintenance, and/or termination. According to the TTM, individuals express stronger commitment to future behavioral change as they move from the precontemplation stage to the stages of contemplation and/or preparation. Individuals considered to be in the action stage actively take steps needed to accomplish the targeted behavioral change. The action stage is said to be terminated when the individual has been practicing the targeted behavior change for approximately six months, after which the individual moves into the maintenance. Prochaska et al. offer ten stage-specific activities or POC to augment one’s passage through the various SOC. Further details of these processes can be found elsewhere [26,27].

According to the TTM, dentists indicating non-assessment of patient HPV infection history/status or not recommending immunization were placed in the ‘pre-action’ stage (precontemplation/ contemplation/ preparation), while those indicating assessing either all or some of their patients’ HPV infection history/status or recommending immunization were included in the ‘action +’ stage (action/ maintenance). Determinants of dentists’ readiness to act in each of the abovementioned categories included demographics, subject knowledge (OPC knowledge, HPV infection knowledge, HPV vaccine knowledge), and other items derived from POC (Table 1). These items evaluate self-perceived barriers to assesses HPV infection history/status as sexually transmitted infection and recommending HPV immunization.

**Table 1:** Processes of change and the corresponding potential self-perceived barriers.

Processes of change	Readiness to assess HPV infection history	Readiness to recommend HPV immunization
	Derived item/statement	Derived item/statement
**Conscious raising**	Knowledge about HPV infection and OPC	Knowledge about HPV vaccine and OPC
**Dramatic relief**	I would not feel comfortable discussing the patient’s sexual history if there is a large age difference (older/younger) between me and the patient. I do not ask about a patient’s sexual history because I worry about confidentiality issues. I do not feel comfortable asking about or discussing a patient’s sexual history.	I am not comfortable discussing sexual history/topics with patients. I am concerned about liability issues. I am concerned about the safety of HPV vaccine.
**Self-reevaluation**	I do not believe it is my role as an oral health provider to discuss sexual topics or issues with any patient.	I do not believe it is my role as an oral health provider to recommend HPV vaccine to my patients.
**Helping relationships**	I would not discuss sexual history with a minor. When discussing sexual history with patient of opposite sex, a staff member of the same sex as patient would also have to be in the room. I would not discuss sexual history with a patient of the opposite sex.	No statement/item was driven from this process of change
**Stimulus control**	The physical layout of my office does not afford enough privacy (e.g., other patients’ presence in a larger exam area, no doors on the exam room, etc.) for me to discuss sensitive topics like sexuality. I do not have enough time to discuss sexual history with a patient.	Dental appointments are not long enough to adequately discuss this topic.

### 2.3 Statistical analyses

Firstly, descriptive analyses were performed: assessed the proportions of respondents as per their socio-demographic characteristics; calculated proportions who were in pre-action or action+ stage for assessing HPV risk and immunization; calculated median overall knowledge score (14 out of 20) and also specific knowledge scores of OPC (2 out of 4), HPV infections (9 out of 10), and HPV immunization (3 out of 6), dichotomized proportion of respondents based on median knowledges scores, and examined their distributions into pre-action or action+ stage; and assessed propotions of agreed or not agreed to statements based on POC and accordingly if they were in pre-action or action+ stage.

Secondly, binary logistic regression analyses were performed to assess the association between each determinant and dentists’ readiness (pre-action vs. action+) to take HPV infection history/status and recommend HPV immunization. Lastly, multivaraible logistic regression models were developed that included only the statistically significant variables (p<0.05) emerging from the simple binary logistic regression models. As 93.13% of the participants reported complete data, all estimates are based on complete-case analyses. The distributions of missing data are reported in S2 File. All statistical analyses were performed using IBM Statistics SPSS 24.

## 3. Results

### 3.1 Descriptive statistics

Of the 9,975 dentists contacted, 932 dentists completed the survey (response rate = 9.3% [932/9,975]). Table 2 provides the respondents’ socio-demographic characteristics. Larger proportions of respondents were male (57.0%), in the age range of 40 to 59 years (50.0%), Canadian trained (61.1%), private general practitioners (84.1%), practicing in urban regions (66.9%), for ten or more years (65.2%). Approximately 76% of respondents affirmed having attended an oral cancer continuing education course within the past 5 years.

**Table 2:** Respondents’ sociodemographic characteristics and their readiness to assess the history of HPV infections and recommend HPV immunization.

Variable	Response	Total (%)	Percentage in the action+ stage to assess history of HPV infections	Percentage in the action+ stage to recommend HPV immunization
Gender	Male	527 (57.0)	53.7%	21.6%
	Female	405 (43.0)	49.7%	18.8%
Age	20–39 years	253 (27.3)	51.2%	16.8%
	40–59 years	463 (50.0)	51.7%	19.7%
	60 years and over	210 (22.7)	52.7%	26.6%
Training country	Canadian trained	553 (61.1)	46.0%	20.4%
	U.S. trained	102 (11.3)	53.0%	19.8%
	IT-DL*	139 (15.4)	65.7%	21.9%
	IT-QP**	111 (12.3)	63.3%	21.8%
Primary Occupation	Private general dentist	781 (84.1)	50.9%	19.0%
	Private specialists	95 (10.2)	51.1%	20.0%
	Other^+^	53 (5.7)	67.3%	44.2%
Practice type	Solo practice	497 (53.6)	51.3%	19.1%
	Group practice	319 (34.4)	51.1%	17.1%
	Solo and group practices	31 (3.3)	61.3%	22.6%
	Public health units clinics	49 (5.3)	64.6%	41.7%
	Private and public health units clinics	32 (3.4)	40.6%	37.5%
Office location	Urban areas	618 (66.9)	53.9%	20.8%
	Suburban/rural areas	306 (33.1)	48.2%	20.0%
Years practicing in Canada	Less than 10 years	322 (34.8)	57.7%	18.2%
	10 or more years	602 (65.2)	48.3%	21.9%
Last time attended continuing education on oral cancer	Within the past year	312 (34.6)	53.1%	23.5%
	In the past 2–5 years	372 (41.3)	51.2%	21.6%
	> 5 years ago	123 (13.7)	56.6%	15.4%
	Never	94 (10.4)	46.9%	14.6%

*IT-DL: Internationally trained dentists practicing through direct licensure.

**IT-QP: Internationally trained dentists practicing after graduating from qualifying program.

^+^ Other includes Uniformed services/Federal employees, residents in a specialty training program, dental school faculty, hospital dentists, and provincial or municipal employees.

**Fig 1:**
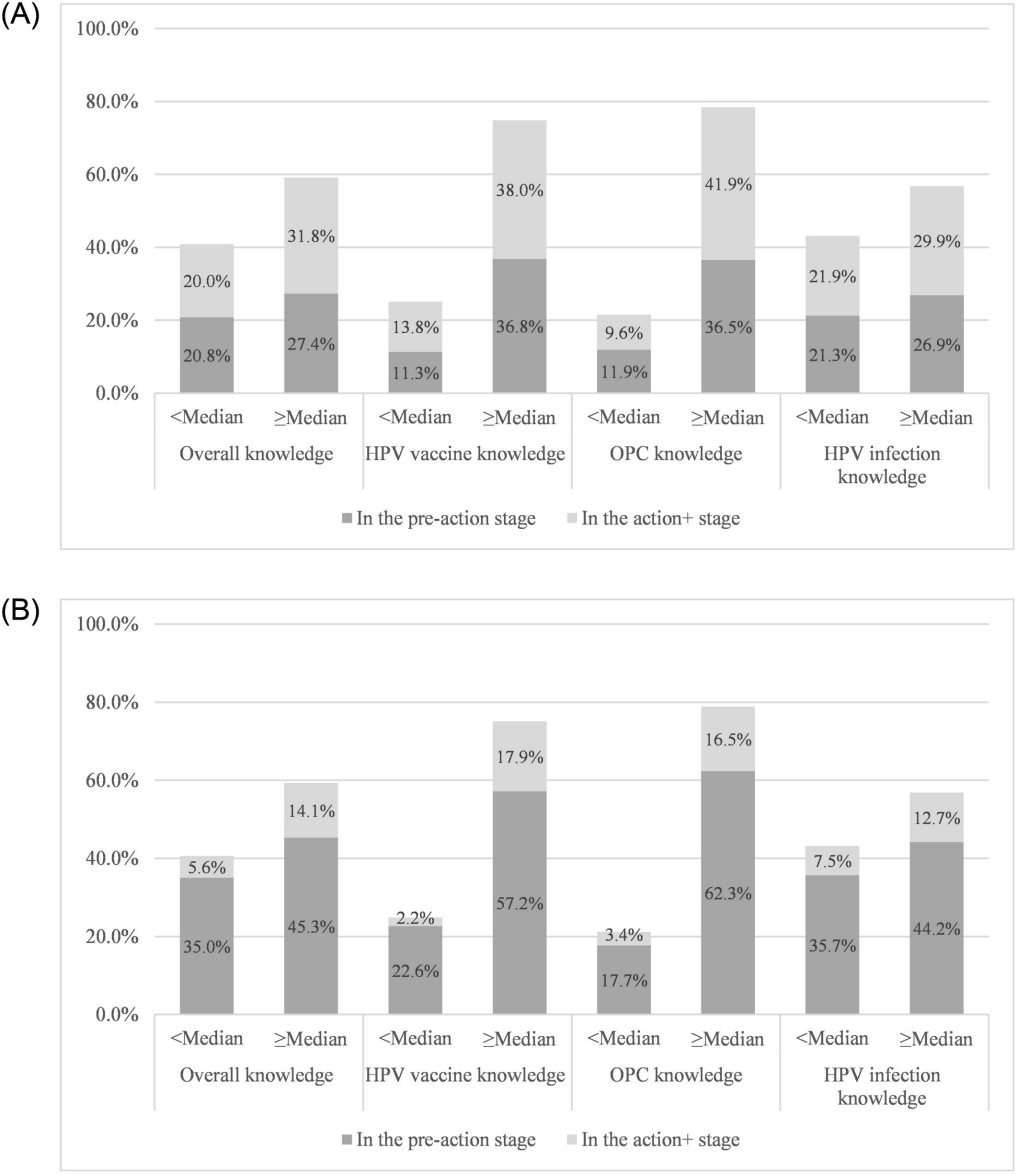
1A. Dentists’ readiness to assess the history of HPV infections according to their knowledge scores. 1B. Dentists’ readiness to recommend HPV immunization according to their knowledge scores.

Approximately 52% of respondents were in the action stage for assessing patient HPV- history/status, but only 20.5% of respondents recommend HPV-immunization to patients (Table 2). Fig 1A and 1B show dentists’ readiness to assess the history of HPV infections and recommend HPV immunization according to their median knowledge scores: overall; OPC; HPV infections; and HPV vaccine. Approximately 60.0% of respondents had equal to or above the median overall knowledge score. However, the proportions varied across the three knowledge domains; 78.5% for OPC, 74.9% for HPV vaccine, and 57.0% for HPV infections.

**Fig 2:**
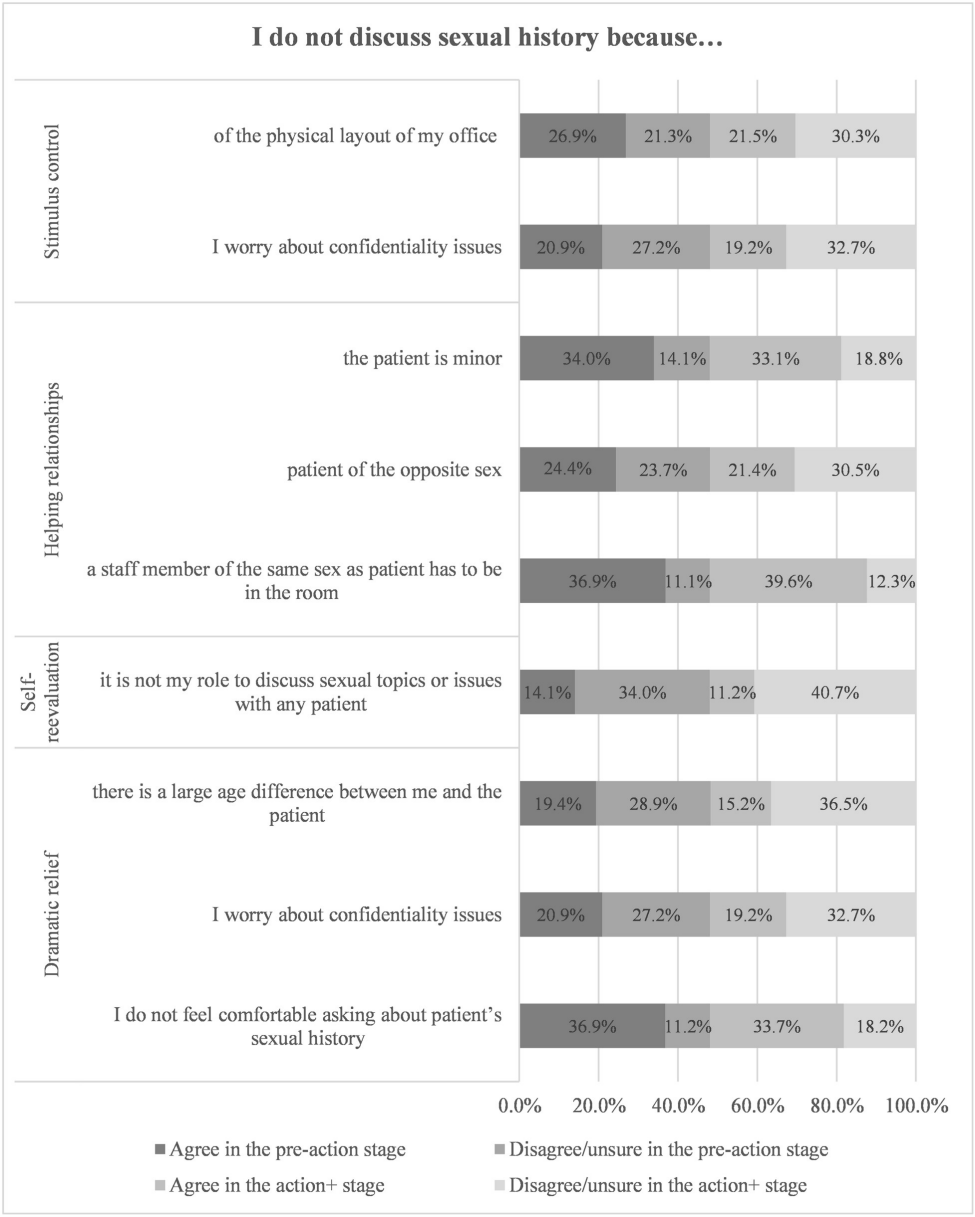
Dentists’ agreements with reasons stated for not discussing sexual history with their patients.

**Fig 3:**
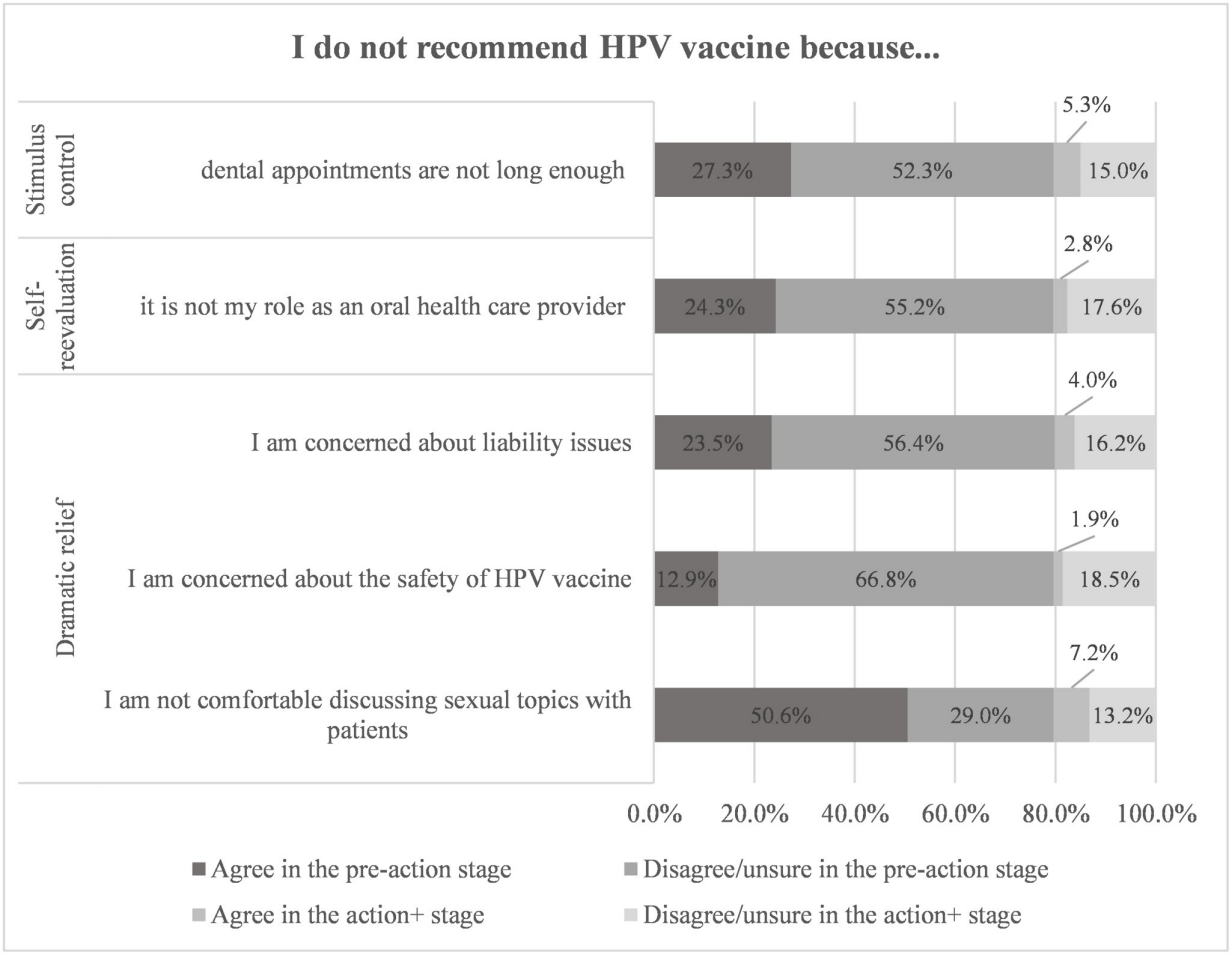
Dentists’ agreements with reasons stated for not recommending HPV immunization.

Fig 2 presents respondents agreement with statements offered as reasons for not discussing patients’ sexual history. The most frequently reported reasons were: 1) the need for a same sex staff member as patient in the operatory (76.5%); 2) discomfort in discussing sex-related topics with patients (70.6%); and 3) perceiving age difference as a barrier with minor patients (67.1%). Fig 3 presents dentists’ reasons for not recommending HPV immunization to patients. The most frequently reported reasons for not recommending HPV immunization were: 1) discomfort in discussing sex-related topics with patients (57.8%); 2) time constraints (32.6%); and 3) self-perceived role (27.1%) of restricting themselves to only oral health related recommendations.

### 3.2 Bivariate models

#### 3.2.1 Readiness to assess history of HPV infections

Statistically significant socio-demographic predictors of dentists’ readiness to assess patient HPV-related history/status include place of training, primary setting of occupation, and years practicing in Canada (Table 3). When compared to Canadian trained dentists, internationally trained practicing through direct licensure (OR = 2.25; 95% CI: 1.52–3.32) or graduated from Canadian dental programs (OR = 2.03; 95%CI: 1.33–3.10) were more likely to be in the action+ stage. Similarly, dentists in non-private settings (OR = 1.99; 95% CI: 1.09–3.61) compared to private general dentists, and dentists practicing for <10 years (OR = 1.46; 95% CI: 1.11–1.92) than those practicing for >10 years were more likely to be in the action+ stage.

**Table 3:** Predictors of dentists’ readiness to assess the history of HPV infections from bivariate and multivariable models.

Variable	Reference	Response	Bivariate model	Multivariable model
			OR (95% CI)	OR (95% CI)
Gender	Female	Male	1.17 (0.90–1.53)	
Age	20–39	40–59 years	1.02 (0.75–1.39)	
		60 years and over	1.06 (0.73–1.53)	
Training country	Canadian trained	U.S. trained	1.33 (0.86–2.03)	1.25 (0.80–1.95)
		IT-DL*	2.25 (1.52–3.32)	2.44 (1.57–3.78)
		IT-Q**	2.03 (1.33–3.10)	2.21 (1.38–3.55)
Primary occupation	Private general dentist	Private specialist	1.01 (0.66–1.54)	1.04 (0.65–1.65)
		Other^+^	1.99 (1.09–3.61)	1.80 (0.92–3.52)
Practice type	Private and public health units clinics	Solo practice	1.54 (0.75–3.19)	
		Group practice	1.53 (0.73–3.20)	
		Solo and group practices	2.31 (0.84–6.35)	
		Public health units clinics	2.67 (1.06–6.69)	
Office location	Suburban/ rural areas	Urban areas	1.26 (0.96–1.66)	
Years practicing in Canada	10 or more years	Less than 10 years	1.46 (1.11–1.92)	1.18 (0.86–1.61)
Last time attended continuing education on oral cancer	Never	Within the past year	1.23 (0.78–1.96)	
		In the past 2–5 years	1.14 (0.73–1.80)	
		> 5 years ago	1.42 (0.83–2.43)	
Overall knowledge scores	< median	≥ median	1.21 (0.92–1.59)	
HPV infections knowledge score	< median	≥ median	1.08 (0.83–1.41)	
OPC knowledge score	< median	≥ median	1.43 (1.04–1.96)	1.51 (1.08–2.14)
HPV vaccine knowledge score	< median	≥ median	0.85 (0.63–1.15)	
Patient of the opposite sex.	Agree	Disagree/unsure	1.48 (1.14–1.92)	1.05 (0.76–1.47)
A staff member of the same sex as patient would also have to be in the room.			0.97 (0.71–1.31)	
Uncomfortable asking about or discussing a patient’s sexual history.			1.77 (1.33–2.37)	1.30 (0.91–1.85)
No enough time to discuss sexual history with a patient.			1.74 (1.30–2.33)	1.39 (1.00–1.94)
I worry about confidentiality issues.			1.31 (1.01–1.71)	1.08 (0.78–1.49)
Minor patients			1.37 (1.04–1.81)	0.90 (0.64–1.27)
Large age difference between me and the patient.			1.61 (1.22–2.12)	1.30 (0.93–1.83)
It is not my role to discuss sexual topics or issues with any patient.			1.50 (1.11–2.03)	1.12 (0.78–1.59)
The physical layout of the clinic			1.78 (1.37–2.31)	1.39 (1.03–1.89)

*IT-DL: Internationally trained dentists practicing through direct licensure.

**IT-QP: Internationally trained dentists practicing after graduating from qualifying program.

^+^ Other includes Uniformed services/Federal employees, residents in a specialty training program, dental school faculty, hospital dentists, and provincial or municipal employees.

In terms of knowledge, dentists whose OPC knowledge scores were equal to or above the median value were more likely (OR 1.43; 95% CI: 1.04–1.96) to assess the history of HPV infections than their counterparts. Statements developed using POC show that dentists who are comfortable with patients of opposite sex and minors, and in discussing sexual history, find dental appointments time adequate, do not worry about confidentiality, perceive assessing HPV history to be in their scope of practice, and do not find their dental office layout as a barrier for such discussions are statistically significantly more in action+ stage than their counterparts.

#### 3.2.2 Readiness to recommend HPV immunization

Statistically significant socio-demographic predictors of dentists’ readiness to recommend HPV vaccine include age, primary setting of occupation, and practice type (Table 4). Older dentists, age 60 years and above, recommended HPV immunization more (OR: 1.79; 95%CI: 1.14–2.82) to their patients than 20–39 year old. Dentists in non-private settings (OR = 3.38; 95% CI: 1.90–6.02) were more likely to recommend than private general dentists. Dentists who practice in solo private practices (OR = 0.39, 95% CI: 0.19–0.83), or in group private practices (OR = 0.34; 95% CI: 0.16–0.74) were less likely to recommend HPV immunization as compared to dentists who practice in both private and public.

**Table 4:** Predictors of dentists’ readiness to recommend HPV immunization from bivariate and multivariable models.

Variable	Reference	Response	Bivariate model	Multivariable model
			OR (95% CI)	OR (95% CI)
Gender	Female	Male	1.19 (0.86–1.65)	
Age	20–39	40–59 years	1.22 (0.81–1.82)	1.23 (0.79–1.92)
		60 years and over	1.79 (1.14–2.82)	1.99 (1.20–3.31)
Training country	Canadian trained	U.S. trained	0.96 (0.57–1.64)	
		IT-DL*	1.09 (0.69–1.72)	
		IT-Q**	1.09 (0.66–1.79)	
Primary occupation	Private general dentist	Private specialist	1.07 (0.63–1.82)	0.88 (0.49–1.59)
		Other^+^	3.38 (1.90–6.02)	0.98 (0.28–3.50)
Practice type	Private and public health units clinics	Solo practice	0.39 (0.19–0.83)	0.44 (0.18–1.09)
		Group practice	0.34 (0.16–0.74)	0.41 (0.16–1.03)
		Solo and group practices	0.49 (0.16–1.47)	0.61 (0.17–2.17)
		Public health units clinics	1.19 (0.48–2.89)	1.03 (0.28–3.81)
Office location	Suburban/rural areas	Urban areas	1.05 (0.75–1.48)	
Years practicing in Canada	10 or more years	Less than 10 years	0.80 (0.56–1.12)	
Last time attended continuing education on oral cancer	Never	Within the past year	1.60 (0.87–2.94)	
		In the past 2–5 years	1.43 (0.78–2.62)	
		> 5 years ago	0.95 (0.45–1.99)	
Overall knowledge scores	< median	≥ median	1.95 (1.35–2.81)	
HPV infections knowledge score	< median	≥ median	1.36 (0.97–1.91)	
OPC knowledge score	< median	≥ median	1.38 (0.90–2.10)	
HPV vaccine knowledge score	< median	≥ median	3.17 (1.94–5.19)	2.96 (1.76–5.00)
I am concerned about the safety of HPV vaccine.	Agree	Disagree/ unsure	1.93 (1.13–3.29)	2.23 (1.11–4.50)
I am concerned about liability issues.			1.70 (1.14–2.54)	0.71 (0.43–1.19)
I do not believe it is my role as an oral health provider to recommend HPV vaccine to my patients.			2.75 (1.76–4.28)	1.92 (1.12–3.27)
Dental appointments are not long enough to adequately discuss this topic.			1.47 (1.03–2.11)	1.02 (0.66–1.56)
I am not comfortable discussing sexual history/topics with patients.			3.20 (2.29–4.47)	2.77 (1.86–4.13)

*IT-DL: Internationally trained dentists practicing through direct licensure.

**IT-QP: Internationally trained dentists practicing after graduating from qualifying program.

^+^ Other includes Uniformed services/Federal employees, residents in a specialty training program, dental school faculty, hospital dentists, and provincial or municipal employees.

In terms of knowledge, dentists scoring equal to or above the median overall knowledge score (OR = 1.95; 95% CI: 1.35–2.81) and HPV immunization knowledge score (OR = 3.17; 95% CI: 1.94–5.19) were more likely to recommend immunization to their patients than their counterparts. Statements based on POC show that dentists who are not concerned about the HPV vaccine safety or liability issues, believe that it is their role to recommend immunization, find dental appointments time adequate, and comfortable discussing sexual history with patients are statistically significantly more in action+ stage than their counterparts.

### 3.3 Multivariable models

#### 3.3.1 Dentists’ readiness to assess history of HPV infections

Place of training, OPC knowledge, and perceiving the physical layout of the clinic as a barrier to assess sexual history remained significant predictors for dentists’ readiness to assess the history of HPV infections (Table 3). Internationally trained dentists through direct licensure (OR: 2.44; 95% CI: 1.57–3.78) or graduated from Canadian dental programs (OR: 2.21; 95% CI: 1.38–3.55) were more likely to assess HPV history among their patients than Canadian trained. Dentists scoring equal to or above the median OPC knowledge score (OR = 1.51; 95% CI: 1.08–2.14), and those who were comfortable with the physical layout of the clinic to discuss the sexual history with their patients (OR = 1.39; 95% CI: 1.03–1.89) were more likely to assess the history of HPV infections than their counterparts.

#### 3.3.2 Dentists’ readiness to recommend HPV immunization

Dentist’s age, HPV vaccine knowledge score, and agreement with statements derived from the POC remained significant predictors for readiness to recommend HPV immunization (Table 4). Dentists, age 60 years and above were more (OR: 1.99; 95% CI: 1.20–3.31) likely to recommended HPV immunization to their patients than 20–39 year olds. Those with higher HPV vaccine knowledge were also more likely (OR: 2.97; 95% CI: 1.76–5.00) to recommend HPV immunization. Dentists who were not concerned with vaccine safety (OR = 2.23; 95% CI: 1.11–4.50), comfortable discussing sexual history with patients (OR = 2.77; 95% CI: 1.86–4.13), and believe that recommending immunization is in their scope of practice (OR = 1.92; 95% CI: 1.12–3.27) were statistically significantly more in action+ stage than their counterparts.

## 4. Discussion

The objectives of this paper were to assess dentists’ readiness to assess the history of patient HPV infections/status and willingness to recommend HPV immunization, and to explore the readiness-associated determinants. Our findings reveal low proportions of dentists in both outcomes, with dentists’ readiness to assess patient HPV history/status higher than their readiness to recommend HPV immunization. However, a comparison of current study findings to earlier findings with Florida dentists, point to an even lower proportion of Florida dentists who were recommending HPV immunization to their female patients [24]. This difference could be related to participants’ characteristics, increase in dentists’ awareness about HPV in the past years, or contextual differences related to access to healthcare and HPV vaccine between the U.S. and Canada. Nonetheless, these findings suggest that dentists can play a key role in minimizing communities’ burden of HPV infection. Dentists are in a unique position to identify and counsel individuals at risk for HPV infection, and those who qualify for HPV immunization. Dentists can also discuss HPV-immunization concerns with patients and monitor vaccine compliance among their patient base.

Dentists’ readiness to assess HPV infection history and status was predicted by place of training, OPC knowledge, and the physical layout of the clinic. The existing literature does not fully explain the difference in readiness to assess history of HPV infections between the Canadian and internationally trained dentists. One possible explanation is that internationally trained dentists are less influenced by the self-perceived barriers because of utilizing more diverse communication techniques [28]. Alternatively, it is possible that Canadian and U.S. trained dentists are facing barriers related to the POC that were not investigated in this study. For example, Shepperd et al (2013) reported that parents’ approval (helping relationships) is a primary source of societal pressure on dentists to provide HPV counseling [29]. Virgolino et al. (2017) reported that healthcare providers face individual and societal barriers in taking sexual history, such as fear of offending patients (environmental re-evaluation), not understanding the importance of taking such history (self-liberation), or inability to communicate effectively (self-efficacy) [30]. Therefore, it is very likely that other POC from the Transtheoretical Model influence dentists’ progress to the action+ stage to assess the history of HPV infections.

In addition to place of training, dentists’ readiness to assess history of HPV infections was also predicted by OPC knowledge, but not HPV infection knowledge. This is understandable as it is expected that educational interventions for dentists would focus primarily on oral implications of HPV infections including OPC. In addition, this finding highlights the importance of more educational interventions to increase OPC related knowledge. Additionally, dentists’ readiness to assess patient history of HPV infection was predicted by whether dentists’ view the physical layout of the clinic as a reason for not assessing the history of HPV infections. This concern was also indicated by a group of dentists and dental hygienists in a mixed-method study [19]; participants were concerned about the lack of privacy due to environmental factors, such as open operatory, as a barrier to discuss the link between HPV and oral cancer with patients. Accordingly, providing dentists with support to facilitate assessing the history of HPV infections, such as standardized medical history forms, should be prioritized to improve dentists’ readiness to assess history of HPV infections [19].

For dentists’ readiness to recommend HPV immunization, among all socio-demographic characteristics, age was the only factor that was positively associated. This finding is consistent with what Hosking et al. (2017) reported when they surveyed a sample of Pediatric Dentistry Graduate Program Directors in the U.S. The increased age among the Program Directors was positively correlated with recommending HPV immunization to patients [31]. The reasons behind observing more acceptance and readiness among older dentists are unknown. It is possible that older dentists are less influenced by the self-perceived barriers as they tend to utilize more communication techniques to overcome their discomfort around recommending HPV immunization [28,32].

In addition to age, dentists’ HPV vaccine knowledge and concerns about the vaccine safety, discussing sex-related topics, and exceeding the scope of practice were significant predictors of dentists’ readiness to recommend HPV immunization. These findings add to the current understanding how dentists’ readiness to recommend HPV is shaped across the SOC. Daley et al (2014) identified that absence of concerns about liability issues is associated with occupying the contemplation stage rather than the pre-contemplation stage while our study suggests that absence of concerns about liability issues does not predict occupying the action+ stage [24]. Daley et al (2014) also recognized that higher knowledge score about HPV vaccine, and absence of concerns about the vaccine safety and discussing sex-related topic do not predict the contemplation, whereas this study demonstrated that these factors predict the action+ stage to recommend HPV immunization [24]. Furthermore, self-perceived role was a significant predictor in Daley (2014) as well as in our study, which suggests that it can predict the occupancy of more than one SOC. Accordingly, it can be proposed that dentists’ readiness to recommend HPV immunization is initially shaped off by the self-perceived role and absence of concerns about liability issues. As dentists maintain the same self-perceived role, HPV vaccine knowledge and absence of concerns about the vaccine safety and discussing sex-related topics are crucial to move dentists to the action+ stage.

Significant predictors identified in this study were derived from four different POC; consciousness raising, dramatic relief, self-reevaluation, and stimulus control. These four processes represent different cognitive and behavioral activities that individuals perform to move between the stages. Consciousness raising and dramatic relief usually mediate transition from pre-contemplation to contemplation. Self-reevaluation often mediates transition from contemplation to preparation, while stimulus control is proposed to mediate transition from action to maintenance [27]. Our results are consistent with these proposed relationships between the processes and stages as dentists who do not experience negative emotions and recognize recommending HPV immunization as part of their scope of practice have moved already from pre-action to action+ stage. This agreement between our results and the proposed relationships implies that moving from pre-action to action+ stage is more likely to be mediated by self-liberation and moving to maintenance is more likely to be mediated by counterconditioning, helping relationship, and reinforcement management. However, readiness to recommend HPV immunization was more aligned with these proposed roles for the POC compared to readiness to assess history of HPV infections, which indicates the need for more research to understand the latter.

Despite the facts that we utilized a theory-informed approach to answer critical research questions, no formal tests have been performed to assess the validity and reliability of the survey instrument. Accordingly, this study should be interpreted as an exploratory study that highlights potential areas for future intervention. The cross-sectional design of our study did not allow us to confirm the mediation effects of the POC. It is possible that these processes were activated after progressing to a given stage. Therefore, caution must be exercised when designing professional interventions to shift dentists to the action+ stage. Moreover, our study did not evaluate all POC due to the length of the survey. Including all the POC and other TTM constructs may change the estimates and/or reveal new predictors. Furthermore, our study dichotomized the SOC into pre-action and action+ stages, which may not directly reveal how dentists move from one particular stage to another stage.

In terms of the generalizability of the study findings to Ontario dentists, the low participation rate in the study may limit the generalizability of the results to Ontario dentists who are similar to the study sample. Although our communication with the RCSO indicates that the characteristics of the study sample is comparable to the characteristics of the registered Ontario dentists in term of age, sex, and training country (data not shown), it is expected that those who participated in the study are those who are currently in the action+, which may overestimate Ontario dentists’ readiness to assess the history of HPV infections and recommend HPV immunization and underestimate the effects of the examined determinants on their readiness [33].

Although this study demonstrated that dentists have the potential to play key roles in the prevention of HPV infections, collaboration between different stakeholders can improve dentist’s readiness to assess the history of HPV infections and recommend HPV immunization. Academic institutions need to enrich their curricula with more information about OPC, HPV immunization and how to communicate such sensitive issues with patients. Regulatory colleges also need to promote developing HPV related content for continuing dental education courses. Professional dental organizations can address dentists’ discomfort and concerns about patients’ privacy by adding items about HPV infections and immunization to the medical history forms. More importantly, the role of dentists in the prevention of HPV infections, including their role in the risk assessment of OPC, and recommending immunization needs to be more clearly defined [34]. Furthermore, the identified concerns should be addressed with respect to the wider context of the barriers and facilitators for oral cancer screening, especially the financial barriers and facilitators in privately financed dental care systems [23]. The potential impact of these proposed intervention are areas for randomized controlled trials. Longitudinal studies are also needed to assess the impact of improving dentists’ readiness on improving their patients’ behaviors regarding HPV, and on minimizing the overall burden of HPV in the community.

## 5. Conclusions

This study shows the minimal contribution dentists are making to the prevention of HPV infection. Also demonstrated is the larger role dentists can play in reducing HPV infection and its sequel. Dentists’ lack knowledge in the areas of HPV-related OPC and available HPV vaccines, including safety and efficacy. Other determinants for dentists’ readiness include patient privacy, discussion of sensitive topic, and dangers of exceeding their scope of practice. Future research should explore the role of the other POC and Transtheoretical Model constructs and patient-related factors in dentists’ readiness to engage in HPV prevention behaviors. Development of educational interventions to ameliorate dentists perceived barriers to HPV screening are also needed.

## Supporting information

S1 File The study questionnaire. (DOCX)

S2 File The distributions of missing data. (DOCX)

## Abbreviations

HPV: Human papilloma virus
OPC: Oropharyngeal cancers
OR: Odds ratio
POC: Processes of change
TTM: Transtheoretical Model
