# Supplementary material for: Determinants of dentists’ readiness to assess HPV risk and recommend immunization: A transtheoretical model of change-based cross-sectional study of Ontario dentists
Source: PLoS One. 2021 Feb 17;16(2):e0247043. doi: 10.1371/journal.pone.0247043 (PMC7888637; doi:10.1371/journal.pone.0247043)
Supplement: S1 File — (DOCX) [file pone.0247043.s001.docx]

**Determinants of Dentists’ Readiness to Assess HPV Risk and Recommend Immunization: A Transtheoretical Model of Change-Based Cross-sectional Study of Ontario Dentists** Musfer Aldossri, Chimere Okoronkwo, Virginia Dodd, Heather Manson, and Sonica Singhal

**S1 File.** **The study questionnaire.**

| 1. Which of the following signs and symptoms can be an indication of early or late stage of oral cancer? (CHECK ONE RESPONSE FOR EACH SIGN/SYMPTOM) | | | | |  |
| --- | --- | --- | --- | --- | --- |
|  |  |  |  |  |  |
|  | Early sign/symptom | Late sign/symptom | Sign/symptom, but not sure early or late | Not a sign/symptom | Don't know |
| Persistent or referred pain |  |  |  |  |  |
| Persistent red and/or white patch |  |  |  |  |  |
| Trismus |  |  |  |  |  |
| Nonhealing ulcer |  |  |  |  |  |
| Small, painful discoloration: red, white or black |  |  |  |  |  |
| Unusual oral bleeding or epistaxis |  |  |  |  |  |
| Dysphagia |  |  |  |  |  |
| Airway obstruction |  |  |  |  |  |
| Paresthesia, dysesthesia of the tongue or lips |  |  |  |  |  |
| Chronic earache (chronic serous otitis media)/ otalgia |  |  |  |  |  |

| 2. Which characteristic(s) describe a patient you would consider at high risk for oral cancer? (CHECK ONE RESPONSE FOR EACH CHARACTERISTIC) | | |  |
| --- | --- | --- | --- |
|  |  |  |  |
|  | Yes | No | Don't know |
| Older age |  |  |  |
| Heavy use of alcohol |  |  |  |
| Use of tobacco products |  |  |  |
| Family history of cancer |  |  |  |
| Low consumption of fruits and vegetables |  |  |  |
| Prior oral cancer lesion |  |  |  |
| Poor fitting dentures |  |  |  |
| Being HPV vaccine recipient |  |  |  |
| Having a history of HPV infection |  |  |  |
| Being a male |  |  |  |
| Being a female |  |  |  |

| 3. To the best of your knowledge, please indicate which of the following statements are true or false. If you are unsure please mark "unsure." | | |  |
| --- | --- | --- | --- |
|  |  |  |  |
|  | True | False | Unsure |
| HPV is a sexually transmitted infection |  |  |  |
| Most sexually active people will contract HPV infection |  |  |  |
| HPV can be transmitted through saliva |  |  |  |
| HPV can cause abnormal pap smears in women |  |  |  |
| HPV can cause genital warts |  |  |  |
| HPV can cause cervical cancer |  |  |  |
| HPV can cause oral cavity cancer |  |  |  |
| HPV can cause oral pharyngeal cancer |  |  |  |
| Oral pharyngeal cancers are more common in men than women |  |  |  |
| Oral pharyngeal cancers are more common in smokers |  |  |  |
| Using a condom decreases the chance of HPV transmission |  |  |  |
| HPV vaccination is recommended at well baby clinics |  |  |  |
| HPV vaccine is recommended for females, 9-26 years of age |  |  |  |
| HPV vaccine is recommended for males, 9-26 years of age |  |  |  |
| HPV vaccine is recommended for females, 26-45 years of age |  |  |  |
| HPV vaccine is recommended for males, 26-45 years of age |  |  |  |
| Oral pharyngeal cancers cannot be detected clinically |  |  |  |
| Oral pharyngeal cancers are only detectable in advanced/late stages |  |  |  |
| There is a government financed HPV vaccine program for boys and girls in grade 7 in Ontario |  |  |  |
| Antibiotics can cure HPV infection |  |  |  |

| 4. When taking a medical history of a new patient, which of the following do you assess? (CHECK ONE RESPONSE ON EACH LINE) | | | |  |
| --- | --- | --- | --- | --- |
|  |  |  |  |  |
|  | Yes, with all patients | Yes, but only with some patients | No, but I have thought about it | No, and have no plans to start |
| Patient's alcohol use |  |  |  |  |
| Patient's tobacco use |  |  |  |  |
| Sexually transmitted infection (STI or STD) history |  |  |  |  |
| Patient’s history of HPV infection |  |  |  |  |
| HPV vaccination status |  |  |  |  |
| Patient’s history of oral cancer |  |  |  |  |

| 5. In your practice, how easy or difficult is it for you to discuss the following topics with your patients? | | | |  |
| --- | --- | --- | --- | --- |
|  |  |  |  |  |
|  | Very easy | Easy | Somewhat easy | Not easy |
| Eating disorders |  |  |  |  |
| Substance abuse (e.g., tobacco use, alcohol use, illicit drugs) |  |  |  |  |
| Sexually transmitted infection (STI/STD) history |  |  |  |  |
| Sexual behaviors (e.g., oral sex, anal sex) |  |  |  |  |
| Physical abuse (e.g., domestic violence, child abuse, elder abuse) |  |  |  |  |

| 6. The table below provides some reasons why dentists might not discuss sexual history with their patients. Please indicate your agreement with each of their statements. | | |  |
| --- | --- | --- | --- |
|  |  |  |  |
|  | Agree | Disagree | Unsure |
| I would not discuss sexual history with a patient of the opposite sex. |  |  |  |
| When discussing sexual history with patient of opposite sex, a staff member of the same sex as patient would also have to be in the room. |  |  |  |
| I do not feel comfortable asking about or discussing a patient’s sexual history. |  |  |  |
| I do not have enough time to discuss sexual history with a patient. |  |  |  |
| I do not ask about a patient’s sexual history because I worry about confidentiality issues. |  |  |  |
| I would not discuss sexual history with a minor. |  |  |  |
| I would not feel comfortable discussing the patient’s sexual history if there is a large age difference (older/younger) between me and the patient. |  |  |  |
| I do not believe it is my role as an oral health provider to discuss sexual topics or issues with any patient. |  |  |  |
| The physical layout of my office does not afford enough privacy (e.g., other patients' presence in a larger exam area, no doors on the exam room, etc.) for me to discuss sensitive topics like sexuality. |  |  |  |

| 7. Please indicate the extent to which each of the following statements reflects your current practice: (CHECK ONE RESPONSE ON EACH LINE) | | | |  |
| --- | --- | --- | --- | --- |
|  |  |  |  |  |
|  | Yes, with all patients | Yes, but with some patients | No, but I have thought about it | No, and have no plan to start |
| I currently discuss with my patients the connection between alcohol and oral cancer. |  |  |  |  |
| I currently discuss with my patients the connection between tobacco and oral cancer. |  |  |  |  |
| I currently discuss with my patients the connection between HPV and oral cancer. |  |  |  |  |
| I currently recommend the HPV vaccine to my patients. |  |  |  |  |

| 8. The table below provides some reasons why dentists might not recommend the HPV vaccine to their patients. Please indicate your level of agreement with these statements. | | |  |
| --- | --- | --- | --- |
|  |  |  |  |
|  | Agree | Disagree | Unsure |
| I am concerned about the safety of HPV vaccine. |  |  |  |
| I am concerned about liability issues. |  |  |  |
| I do not believe it is my role as an oral health provider to recommend HPV vaccine to my patients. |  |  |  |
| Dental appointments are not long enough to adequately discuss this topic. |  |  |  |
| I am not comfortable discussing sexual history/topics with patients. |  |  |  |

9. Please indicate how often you provide an oral cancer examination for each patient age group at complete exam and recall exam. Please select a response from each drop down menu.

|  |  |  |
| --- | --- | --- |
| Age | Complete exam | Recall exam |
| 0-8 |  |  |
| 9-26 |  |  |
| 27-45 |  |  |
| 46-64 |  |  |
| 65+ years |  |  |

10. When performing oral cancer exams, which of the following sites do you routinely check? (CHECK ALL THAT APPLY)

Soft palate

Hard palate

Anterior two thirds of tongue

Posterior one third of tongue

Gingiva

Buccal mucosa

Floor of mouth

Tonsils

Lymph nodes

Pharynx

None of the above

| 11. Please indicate your confidence level when using the following oral cancer screening tools: | | | | |  |
| --- | --- | --- | --- | --- | --- |
|  |  |  |  |  |  |
|  | Very confident | Confident | Somewhat confident | Not confident | Have never used |
| Visual inspection |  |  |  |  |  |
| Radiographs (OPGs, CBCT) |  |  |  |  |  |
| Palpation/manual examination of tongue, cheeks, and neck |  |  |  |  |  |
| Toluidine Blue (TB) |  |  |  |  |  |
| Light detection (e.g., ViziLite, VELscope) |  |  |  |  |  |
| Exfoliative cytology |  |  |  |  |  |
| Brush biopsy (e.g., OralCDx ) |  |  |  |  |  |

| 12. The statements below reflect issues which might interfere with the ability to regularly provide oral cancer exams to patients. Please indicate your level of agreement with each statement. Please indicate "Not applicable" if the statement does not apply to you. | | |  |
| --- | --- | --- | --- |
|  |  |  |  |
|  | Yes | No | Not applicable |
| No separate code/fees associated with oral cancer exam. |  |  |  |
| Other dentists do not commonly provide routine oral cancer exams. |  |  |  |
| Routine oral cancer exam is not necessary for each patient. |  |  |  |
| Professional dental organizations have not provided clinical guidelines for oral cancer exams. |  |  |  |
| Oral cancer exam take too much time. |  |  |  |
| My knowledge about oral cancers is not current. |  |  |  |
| I do not feel confident in my ability to perform an adequate oral cancer exam. |  |  |  |
| I am not comfortable palpating lymph nodes in patient's neck. |  |  |  |
| I am uncomfortable discussing oral cancer risk factors with patients. |  |  |  |
| Oral cancer examinations cause patients too much concern. |  |  |  |

13. Does one or more dental hygienists employed by your office provide oral cancer screening for patients?

Yes

No

Don’t know

I do not have a dental hygienist in my office

| 14. Do any of the following factors will increase your likelihood of providing regular oral cancer examinations to each of your patients? | |  |
| --- | --- | --- |
|  |  |  |
|  | Yes | No |
| A separate code in the suggested fee guide for oral cancer exam. |  |  |
| More training on how to perform oral cancer exams. |  |  |
| More time available for patient exam. |  |  |
| Availability of more efficient screening tools. |  |  |
| Availability of less expensive screening tools. |  |  |

| 15. Please indicate the extent to which you personally agree or disagree with each of the following statements: | | | | |  |
| --- | --- | --- | --- | --- | --- |
|  |  |  |  |  |  |
|  | Strongly Agree | Agree | Disagree | Strongly Disagree | Don’t know |
| I am adequately trained to provide tobacco cessation counseling. |  |  |  |  |  |
| I am adequately trained to assess usage and provide alcohol cessation counseling. |  |  |  |  |  |
| I am adequately trained to recommend the HPV vaccine to patients. |  |  |  |  |  |
| I am adequately trained to recognize the early signs and symptoms of oral cancer. |  |  |  |  |  |
| I am adequately trained to take biopsy samples from suspected oral cancer sites. |  |  |  |  |  |

16. Please indicate your age group

20-29 years

30-39 years

40-49 years

50-59 years

60 years and over

17. Please indicate your gender

Male

Female

Other:Other:

18. What is your primary occupation? (CHOOSE ONLY ONE)

Private practice general dentist

Private practice specialist

Dental school faculty/staff member

Uniformed services/Federal employee

Provincial or municipal employee

Hospital staff dentist

Graduate student/resident in a speciality training program

Other:Other:

19. Which of the following describes your practice setting(s)? (CHECK ALL THAT APPLY)

Solo private practice (owned)

Solo private practice (associate)

Group private practice (owned)

Group private practice (associate)

Community health center/ public health unit

Armed forces

Dental school clinics

Hospital

Other:Other:

20. Your main office/practice is located in:

Urban area

Sub-urban area

Rural area

21. You are:

Canadian trained

U.S. trained

Internationally trained with direct licensure

Internationally trained and attended a qualifying program before licensure

Prefer not to answer

22. Please indicate the year you received your primary dental degree (irrespective of the graduation country):

23. Please indicate for how many years you have been practicing in Canada?

Less than one year

1 to < 3 years

3 to < 5 years

5 to < 10 years

10 or more years

24. When was the last time you attended a continuing education course on oral cancer?

Within the past year

During the past 2-5 years

More than 5 years ago

Never

Have yet to attend

Don’t know

25. What are your format preferences for continuing dental education? (RANK YOUR THREE MOST PREFERRED FORMATS BY DRAGGING AND DROPPING IN ORDER (1,2,3))

Handout/booklet with self-test

Continuing education journals

Online training modules

Webinars including clinical demonstration

Lecture/Hands-on sessions organized by academic institutes

Hands-on sessions organized by diagnostic aids manufacturers

Lecture/Hands-on sessions organized by Ontario Dental Association (ODA) or its regional partners
