# Supplementary material for: Determinants of dentists’ readiness to assess HPV risk and recommend immunization: A transtheoretical model of change-based cross-sectional study of Ontario dentists
Source: PLoS One. 2021 Feb 17;16(2):e0247043. doi: 10.1371/journal.pone.0247043 (PMC7888637; doi:10.1371/journal.pone.0247043)
Supplement: S2 File — (DOCX) [file pone.0247043.s002.docx]

**Determinants of Dentists’ Readiness to Assess HPV Risk and Recommend Immunization: A Transtheoretical Model of Change-Based Cross-sectional Study of Ontario Dentists** Musfer Aldossri, Chimere Okoronkwo, Virginia Dodd, Heather Manson, and Sonica Singhal

**S2 File. The distributions of missing data.**

| Variable | All (n=932) | HPV history (n=932) | | | HPV immunization (n=932) | | |
| --- | --- | --- | --- | --- | --- | --- | --- |
|  |  | Pre-action stage (n=442) | Action+ stage (n=477) | Missing (n=13) | Pre-action stage (n=726) | Action+ stage (n=194) | Missing (n=12) |
| Gender (n=924) | 8 | 4 | 2 | 2 | 5 | 1 | 2 |
| Age (n=926) | 6 | 1 | 3 | 2 | 1 | 3 | 2 |
| Training country (n=905) | 27 | 12 | 13 | 2 | 21 | 4 | 2 |
| Primary Occupation (n=929) | 3 | 0 | 1 | 2 | 1 | 0 | 2 |
| Practice type (n=928) | 4 | 1 | 1 | 2 | 1 | 0 | 3 |
| Office location (n=924) | 8 | 4 | 2 | 2 | 5 | 1 | 2 |
| Years practicing in Canada (n=924) | 8 | 0 | 6 | 2 | 6 | 0 | 2 |
| Last time attended continuing education on oral cancer (n=901) | 31 | 17 | 12 | 2 | 26 | 3 | 2 |

**S2-A Table. The distribution of missing data across sociodemographic variables and stages of changes.**

**S2-B Table. The distribution of missing data across the perceived barriers to evaluate patients’ sexual history.**

| Variable | All (n=932) | HPV history (n=932) | | |
| --- | --- | --- | --- | --- |
|  |  | Pre-action stage (n=442) | Action+ stage (n=477) | Missing (n=13) |
| Patient of the opposite sex. | 9 | 1 | 1 | 7 |
| A staff member of the same sex as patient would also have to be in the room. | 10 | 2 | 2 | 6 |
| Uncomfortable asking about or discussing a patient’s sexual history. | 8 | 1 | 1 | 6 |
| No enough time to discuss sexual history with a patient. | 10 | 3 | 1 | 6 |
| I worry about confidentiality issues. | 13 | 3 | 4 | 6 |
| Minor patients | 10 | 2 | 2 | 6 |
| Large age difference between me and the patient. | 11 | 1 | 4 | 6 |
| It is not my role to discuss sexual topics or issues with any patient. | 9 | 2 | 1 | 6 |
| The physical layout of the clinic | 9 | 1 | 2 | 6 |

**S2-C Table. The distribution of missing data across the perceived barriers to evaluate recommend HPV immunization.**

| Variable | All (n=932) | HPV immunization (n=932) | | |
| --- | --- | --- | --- | --- |
|  |  | Pre-action stage (n=726) | Action+ stage (n=194) | Missing (n=12) |
| I am concerned about the safety of HPV vaccine. | 10 | 3 | 0 | 7 |
| I am concerned about liability issues. | 18 | 4 | 6 | 8 |
| I do not believe it is my role as an oral health provider to recommend HPV vaccine to my patients. | 8 | 0 | 1 | 7 |
| Dental appointments are not long enough to adequately discuss this topic. | 10 | 0 | 3 | 7 |
| I am not comfortable discussing sexual history/topics with patients. | 11 | 2 | 2 | 7 |
